# A randomized controlled trial investigating the effect of Pycnogenol and *Bacopa *CDRI08 herbal medicines on cognitive, cardiovascular, and biochemical functioning in cognitively healthy elderly people: the Australian Research Council Longevity Intervention (ARCLI) study protocol (ANZCTR12611000487910)

**DOI:** 10.1186/1475-2891-11-11

**Published:** 2012-03-06

**Authors:** Con K Stough, Matthew P Pase, Vanessa Cropley, Stephen Myers, Karen Nolidin, Rebecca King, David Camfield, Keith Wesnes, Andrew Pipingas, Kevin Croft, Dennis Chang, Andrew B Scholey

**Affiliations:** 1 Centre for Human Psychopharmacology, Swinburne University of Technology, Melbourne, Australia; 2 Melbourne Neuropsychiatry Centre, The University of Melbourne, Australia; 3 Southern Cross University, Lismore, Australia; 4 CDR, Bracket, Goring-On-Thames, England; 5 School of Medicine and Pharmacology, University of Western Australia, Australia; 6 Complemed, NICM, University of Western Sydney, Australia

**Keywords:** Pycnogenol, Bacopa, CDRI08, Pine bark, Brain, Dementia, Cognitive, Cognition, Ageing, RCT

## Abstract

**Background:**

One of the major challenges associated with our ageing population is the increasing incidence of age-associated cognitive decline, which has significant implications for an individual's ability to lead a productive and fulfilling life. In pure economic terms the costs of ageing reflects decreased productivity and engagement with the workforce. The maintenance of brain health underpinning intact cognition is a key factor to maintaining a positive, engaged, and productive lifestyle. In light of this, the role of diet, including supplementation with nutritional and even pharmacological interventions capable of ameliorating the neurocognitive changes that occur with age constitute vital areas of research.

**Methods:**

In order to reduce cognitive ageing, the ARC longevity intervention (ARCLI) was developed to examine the effects of two promising natural pharmacologically active supplements on cognitive performance. ARCLI is a randomized, placebo-controlled, double-blind, 3-arm clinical trial in which 465 participants will be randomized to receive an extract of *Bacopa monnieri *(CDRI08 300 mg/day), Pycnogenol (150 mg/day), or placebo daily for 12 months. Participants will be tested at baseline and then at 3, 6 and 12 months post-randomization on a wide battery of cognitive, neuropsychological and mood measures, cardiovascular (brachial and aortic systolic and diastolic blood pressures as well as arterial stiffness), biochemical (assays to measure inflammation, oxidative stress and safety) as well as genetic assessments (telomere length and several Single Nucleotide Polymorphisms). The primary aim is to investigate the effects of these supplements on cognitive performance. The secondary aims are to explore the time-course of cognitive enhancement as well as potential cardiovascular and biochemical mechanisms underpinning cognitive enhancement over the 12 months of administration.

ARCLI will represent one of the largest and most comprehensive experimental clinical trials in which supplements are administered to elderly participants. Results from ARCLI may help develop novel preventative health practices and nutritional/pharmacological targets in the elderly for cognitive and brain health.

**Trial registration:**

Australia and New Zealand Clinical Trials Register (ANZCTR): ACTRN12611000487910

## Background and Rationale

With increasing life expectancies and the maturation of the "baby boom" generation, adapting to the challenges posed by the ageing population has been identified as one of the major issues facing contemporary society [1]. Human ageing has significant societal, economic, health and, importantly, personal costs. In pure economic terms the costs of ageing reflects decreased productivity as well as increased levels of reliance on public services for health and social support but this also has obvious ramifications for older people's ability to lead fulfilling lives. Increasing age is associated with a cluster of illnesses many involving oxidative stress and low level chronic inflammation. These include cardiovascular and respiratory disease and, importantly neurological conditions such as Parkinson's disease (PD) and Alzheimer's disease (AD).

### What is Cognitive Ageing?

Individual age-related changes in cognition vary greatly. However research in cognitive aspects of ageing (typically in 60 to 90 year-olds) has identified consistent deficits in: reasoning and decision making; spatial abilities; perceptual-motor and cognitive speed; and most robustly memory (e.g. [2]). Longitudinal studies of aged populations illuminate the time-course of cognitive deterioration. Using 5 to 10 year re-test intervals significant decrements across most cognitive capacities become evident. A recent review of longitudinal ageing studies concludes that crystallized intelligence (e.g., factual knowledge) remains intact until late ageing whereas measures of speed, information processing and aspects of memory (e.g., working memory) are more sensitive to decline from age 60 [2].

### Brain Ageing and Oxidative Stress

Neuroimaging studies reveal that increasing age is reliably associated with ventricular enlargement, reduction in gross brain volume, reductions in frontal and temporo-parietal brain volume, higher levels of cortical atrophy, and increased white matter hyperintensities [3]. Ultimately, shrinkage of cortical volume reduces cognitive capacity [4] and age-related increases in neuropathological events such as beta-amyloid protein deposition and formation of neurofibrillary tangles represent significant risk factors for cognitive decline and AD. Neuropathological events such as beta-amyloid deposition are not exclusive to neurodegenerative disorders such as AD, in fact occurring in a large proportion of cognitively intact individuals. For example, in one study the proportion of non-clinical subjects with beta-amyloid deposits ranged from 3% in a 36-40 age group to 75% in a 85+ age group [5].

Alongside age-associated cortical degeneration [4], there exist numerous microscopic insults related to oxidative stress and free radical damage. Free radicals are molecules with unpaired electrons. These molecules are highly unstable and can cause damage to proteins, lipids, carbohydrates and nucleotides [6]. Free radicals formed in the brain produce significant cellular damage, and mediate processes which result in neural cell death on large scales [7]. Between 95% and 98% of free radicals and Reactive Oxygen Species (ROS) (O_2_^•-^, HO^•^, H_2_O_2_) are formed by mitochondria as by-products of cellular respiration. Studies of mitochondria isolated from the brain show that 2-5% of total oxygen consumed yields ROS [8], these highly reactive molecules make a significant contribution to the peroxidation of principal cell structures (e.g. membrane lipids) [8]. Brain tissue is particularly susceptible due to it's disproportionately high metabolic rate and levels of oxygen, the cytotoxic actions of glutamate, and it's high concentrations of peroxidisable unsaturated fatty acids [7]. Ageing decreases the brain's ability to combat the actions of free radicals and is associated with increased levels of pro-oxidant mediators and decreased antioxidant levels [9]. The relationship between cognition and oxidative stress is evident in the extensive damage caused by free radicals in age-related neurological conditions [10,11], and animal models of age-related oxidative injury with central cognitive and behavioural impairments [12]. Concurrent with the normal age-related cognitive changes are increases in the formation of brain ROS resulting in significant damage to DNA, proteins and in particular membrane lipids [13]. Although multiple factors precipitate oxidative stress throughout the body, the brain is particularly vulnerable and its cumulative effects may account for the delayed onset and progressive nature of Alzheimer's and Parkinson's dementias, as well as normal age-related mental deterioration [10].

### Antioxidants and Cognition

The central role of oxidative stress in age-related cognitive decline and neurodegenerative diseases has driven numerous studies examining the potential benefits of antioxidants in altering, reversing, or forestalling neuronal and behavioural changes (e.g. [14]). Anti-oxidant supplementation results in improved cognition and behaviour in aged animals and concurrent decreases in oxidative insult to neural structures [15]. Human research in this area is largely limited to epidemiological studies. These have identified positive associations in aged individuals between biological levels of dietary antioxidants (vitamins E and C) and working memory measures including the Wechsler Memory test [16]. Less reliable than biological measures, large scale studies (3000+ participants) have also identified positive relationships between dietary intake of vitamin C and E and standardized memory measures [17]. While these non-clinical trials do not demonstrate causality, the consensus that memory is the main cognitive variable affected by antioxidant status is consistent with patterns of age-related cognitive decline and the *in vivo *neuroanatomy of lipid peroxidation [18].

Given the consequences of a burgeoning ageing population, the role of supplementation with nutritional and pharmacological interventions capable of ameliorating the neurocognitive changes that occur with age constitute vital areas of research.

### Treatments to Reduce or Ameliorate Brain Ageing

**(i) Pycnogenol **Neural cells have three systems of protection and repair in response to oxidative stress; 1) Enzymatic antioxidants (superoxide dismutase, catalase, peroxidases); 2) Radical scavenging lipid-soluble (tocopherols, **flavonoids**, carotenoids) and water- soluble (ascorbate, glutathione) antioxidants; and 3) repair proteases and phospholipases [7]. Pycnogenol^® ^is a patented concentrate consisting of condensed flavonoids isolated from Maritime Pine bark. Extensive reviews by Packer et al [19] and Rohdewald [20] have established the antioxidant activity of Pycnogenol in simplified assay systems, cultured cell models, and perfused organs. There is evidence that Pycnogenol prolongs the lifetime of the ascorbate antioxidant and stimulates the synthesis of antioxidant enzymes inside arterial cells [21]. The established neuroprotective properties of flavonoids strongly advocate their use in intervention strategies [22]. Of these, Pycnogenol is particularly promising, having been studied extensively in the context of protecting against oxidative damage to specific neural systems. Kobayashi [23] reported that Pycnogenol had neuroprotective properties in HT-4 neuronal cells subjected to glutamate-induced cytotoxicity (one of the principle sources of ROS in the brain [10]. Similarly Liu [24] and Peng [25] found Pycnogenol to inhibit beta-amyloid apoptosis of neurons and vascular cells, both of which are oxidative processes that accumulate with age, and are pathological features of AD. In vivo supplementation to aged animals [26] results in biological and behavioural changes consistent with the slowing of age-related decline in physiology, learning and memory. Additionally 6 wk Pycnogenol supplementation to humans resulted in significantly reduced serum low density lipoprotein and increased plasma oxygen radical absorbance capacity (ORAC) [27] - a convenient although non-specific measure of in vivo antioxidant activity [28].

**(ii) Bacopa Monniera **(L.) Wettst. (syn. *Bacopa monniera *Hayata & Matsum) has been used in traditional Ayurvedic medicine for various indications including memory decline, inflammation, pain, pyrexia, epilepsy and as a sedative [29]. *Bacopa *contains Bacoside A and Bacoside B which are steroidal saponins believed to be essential for the clinical efficacy of the product. While *Bacopa *has been reported to have many actions, its memory enhancing effects have attracted most attention and are supported by the psychopharmacology literature. Behavioural studies in animals have shown that *Bacopa *improves motor learning, acquisition and retention, and delay extinction of newly acquired behaviour [30]. Although the exact mechanisms of action remain uncertain, evidence suggests that *Bacopa *may modulate the cholinergic system and/or have antioxidant and metal chelating effects [31,32]. *Bacopa *may also have anti inflammatory [33], anxiolytic and antidepressant actions [34,35], relaxant properties in blood vessels [36] and adaptogenic activity [37]. Chronic administration of *Bacopa *inhibits lipid peroxidation in the prefrontal cortex, striatum and hippocampus via a similar mechanism to vitamin E [38]. In an animal model of AD, there was a dose-related reversal by *Bacopa *of cognitive deficits produced by the neurotoxins colchicine and ibotenic acid [32]. In rodents, *Bacopa *inhibited the damage induced by high concentrations of nitric oxide in astrocytes [39]. Memory deficits following cholinergic blockade by scopolamine were reversed by *Bacopa *treatment. In animal studies *Bacopa *reduced lipid peroxidation induced by FeSO_4 _and cumene hydroperoxide indicating that, similarly to the chelating properties of EDTA, it acts at the initiation level by chelating Fe^++ ^[40]. More recently in transgenic mice, *Bacopa *supplementation reduced specific amyloid peptides by up to 60% whilst also improving memory performance [41]. Thus, *Bacopa *appears to have multiple modes of action in the brain all of which may be useful in ameliorating cognitive decline in the elderly. These include: (i) direct pro-cholinergic action; (ii) anti-oxidant (flavonoid) activity; (iii) metal chelation; (iv) anti-inflammatory effects; (v) improved blood circulation; (vi) adaptogenic activity; and (vii) removal of β-amyloid deposits. In contrast to anti-oxidants such as Vitamin C and E, and specific anti-oxidants such as Pycnogenol, the polypharmacological actions of *Bacopa *potentially act on several pathological changes in elderly brains in concert. This potential is supported by a limited number of clinical trials into the neurocognitive effects of Pycnogenol and, to a greater extent, Bacopa. Both substances are capable of improving memory functioning in cognitively intact cohorts, with Pycnogenol improving working memory [42] and *Bacopa *preferentially enhancing secondary memory (reviewed in Pase et al.[43]). In both cases effects are evident at three months of daily administration, but not earlier.

## Design and methodology

### Design

ARCLI is a randomized, double-blind, placebo-controlled, 3-arm parallel-groups clinical trial with participants randomized to receive *Bacopa*, Pycnogenol or a placebo.

### Aims and study hypotheses

Extending upon preliminary findings [42,44,45], the primary aim of the current study is to examine the individual chronic 12 month effects of *Bacopa *(300 mg daily), and Pycnogenol (150 mg daily) on cognitive performance in a healthy elderly population. The secondary aims of the current study are two fold. Firstly, to investigate the time course of cognitive ehancement with follow-up testing at 3, 6 and 12 months. Secondly, to examine putative mechanisms underpinning any cognitive enhancing actions of the supplements by examining relationships between cognitive, biological (biochemical and genetic) and cardiovascular variables over the 12 months. By examining the interrelationship between inflammation, oxidative stress, cardiovascular health and cognitive perfromance, the current study aims to identify modifiable risk factors for cognitive decline that can be targeted by supplementation. It is hypothesized that all supplements will improve measures of cognitive performance, relative to placebo, at all time points with the greatest cognitive effects observed at 12 months.

### Centres

ARCLI will be conducted at the Centre for Human Psychopharmacology, Swinburne University, Melbourne, Australia. In the first year approximately 1/3 of the cohort will be enrolled. In years 2 and 3 additional recruitment will occur at the University of Western Sydney (Sydney, Australia).

### Participants

A total of 465 healthy, elderly participants aged between 60 and 75 years will take part in the study. This restricted age range was chosen due to the large variation in cognitive abilities and trajectories associated with ageing. Participants will be randomized to receive one of three daily treatments for 12 months: ***(a) ***300 mg *Bacopa *(KeenMind CDRI 08 extract); ***(b) ***150 mg Pycnogenol; or (c) placebo. Participants will be excluded from participation if they are a current smoker; have a psychiatric or neurological disease; significant endocrine, gastrointestinal or cardiovascular disorder; other disorder affecting food metabolism; recent history (past 5 years) of chronic/severe illness (longer than 6 weeks); current regular alcohol use exceeding 14 standard drinks per week for women and 28 standard drinks per week for men; vision that is not corrected to normal. To be eligible, participants cannot be taking psychoactive medication including, antidepressants, antipsychotics, anxiolytics, cholinesterase inhibitors, illicit drugs or significant cognitive enhancing drugs (e.g. chronic intake of substances such as Ginkgo). Participants who are irregular users of vitamin or herbal supplements will be asked to stop taking them for the duration of the trial. Participants who are regular users (defined as daily intake for greater than 3 months) of vitamins or herbal supplements will be asked to maintain the same habits throughout the trial. Participants with either global cognitive impairment or significant levels of depressive symptoms, as determined by a score < 24 on the Mini Mental State Examination (MMSE) or a score > 19 on the Geriatric Depression Scale respectively, will be excluded. To ensure that participants do not have probable dementia, those scoring between 24-26 on the MMSE will be administered the Dementia Rating Scale II (DRS-II). Any questionable cases on the DRS-II will be discussed by the ARCLI participant safety committee for agreement on eligibility. The study was ethically approved by the Swinburne University Human Research Ethics Committee (project number 2010/106) and all participants will provide written informed consent. The trial has been registered with the Australian and New Zealand Clinical Trials Registry (ACTRN12611000487910).

### Procedure

**Figure 1:**
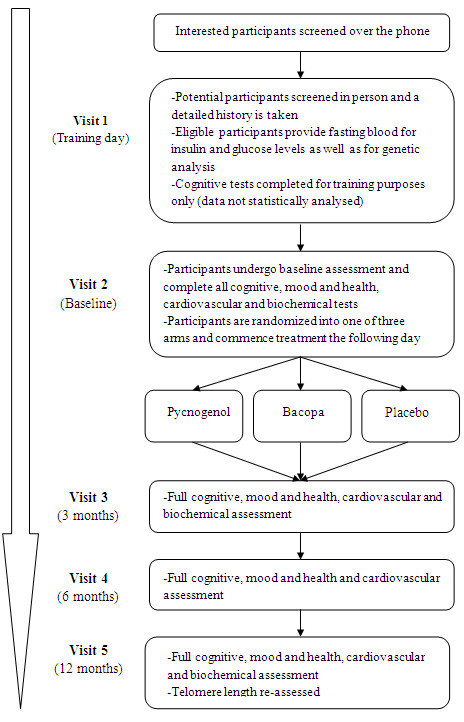
**ARCLI protocol flow diagram**.

Eligible participants are required to attend five testing session. An overview of the testing sessions is provided in the clinical trial flow chart (Figure 1). During the first session, participants are screened for eligibility and a detailed history is taken. Eligible participants are then asked to give blood and to complete the cognitive test batteries multiple times. This allows the participant to familiarize themselves with the cognitive tasks and minimises practice effects (as well as establishing that the participant lies within established norms for their age group). Cognitive data obtained during the training visit will not be included in statistical analysis. Visit 2 involves baseline assessment of all measures and randomization to treatment. Participants are to commence taking their assigned treatment on the day following their baseline assessment. As depicted in Figure 1, visits 3 to 5 involve follow-up assessment at 3, 6 and 12 months post randomization. At the 12 month assessment, participants will be asked to return any remaining supplements, such that they can be counted enabling the investigators to estimate compliance to treatment.

### Sample size

The sample size for this study is 465 participants with 155 participants in each arm. Previous studies using Bacopa and Pycnogenol have reported statistical significance on measures of cognition with approximately 100 participants in each of these studies respectively [42,44]. Based on these previous studies, the current sample size of 465 ensures adequate power to detect a significant effect 80% of the time when conducting two-tailed tests using 95% confidence even when allowing for a 10-20% attrition rate.

### Treatments

(a) The Bacopa supplement used in ARCLI is an extract of *Bacopa *monnieri called CDRI08 and is commercially available as KeenMind™ (Flordis). This product is manufactured from the stems, leaves and roots of *Bacopa *and is extracted with 50% ethanol. It is standardized to contain active bacosides at levels of 55% ± 5%. This extract has previously been shown to enhance cognitive performance at this dosage after 3 months of supplementation [44,45].

(b) Pycnogenol (Horphag Research, Geneva, Switzerland) is a patent extract made exclusively from the bark of French maritime pine trees (*Pinus pinaster*). Pycnogenol contains consistent proportions of bioflavonoids and is standardized to contain 70 ± 5% procyanidins. This dosage of Pycnogenol has previously been shown to enhance memory performance after 3 months of supplementation [42].

### Randomization and safety

An independent researcher (Medical practitioner) located at an independent university will be responsible for the randomization of the study treatments and will chair the safety committee. Participants will be randomly assigned to one of the three treatment groups according to a Latin square design. Randomization codes will be kept in a sealed opaque envelope in a secure safe and will only be opened in case of emergency.

### Primary outcome

The primary study outcome is the effect of supplementation on cognitive performance as measured by a battery of well validated and highly sensitive cognitive tests. These tests will be implemented at baseline and all follow-up time points (3, 6 and 12 months post baseline). This battery will include all tasks from the Cognitive Drug Research (CDR) Computerized Assessment System and selected tasks from the Swinburne University Computerized Cognitive Assessment Battery (SUCCAB). Table 1 lists the cognitive measures assessed by the CDR and SUCCAB batteries. Wechsler intelligence (Wechsler Abbreviated Scale of Inteligence-WASI) will also be administered as both an estimate of pre-morbid intelligence as well as a factor to determine whether IQ predicts response to any of the supplements.

**Table 1:** Cognitive domains measured by CDR and SUCCAB tests

CDR	SUCCAB	Hick RT	Inspection Time
Simple RT	Stroop Colour-Word	Simple Decision Time	Perceptual Speed
Choice RT	Spatial Working Memory	Simple Movement Time	
Spatial WM	Contextual Memory	2 Choice Decision Time	
Numeric WM	Immediate/Delayed Recognition	2 Choice Movement Time	
Picture Presentation		4 Choice Decision Time	
Immediate Word Recall		4 Choice Movement Time	
Digit Vigilance		8 Choice Decision Time	
Delayed Word Recall		8 Choice Movement Time	
Word Recognition			
Picture Recognition			
Rapid Visual Information Processing			

CDR = Cognitive Drug Research, SUCCAB = Swinburne University Computerized Cognitive Assessment Battery, RT = Reaction Time, WM = Working Memory

The CDR battery has been used in well over 1000 clinical trials world wide, is well validated, and has been shown to be sensitive to the effects of several natural supplements including Bacopa and Pycnogenol [42,44]. The SUCCAB is sensitive to the effects of age-associated cognitive decline and has been recommended for use when assesing cognitive changes following nutraceutical supplementation [46]. The SUCCAB has also been shown sensitive to the effects of a pine bark extract similar to Pycnogenol [47]. Both the CDR and SUCCAB provide detailed assessments of memory performance (both short and long term), a domain of cognition expected to be modulated by treatment. The cognitive demand battery [48] will be implemented to assess the effects of supplementation on cognitive effort and fatigue whilst the MMSE [49] will be used as a dementia screening tool at intake and to monitor the effects of treatment on global cognitive decline (after randomization). The Inspection Time task [50] and Hick Reaction Time paradigm [51] will be used as they provide highly valid and sensitive assessment of mental speed which has been shown to slow with increasing age.

### Secondary outcomes

A wide range of psychological, cardiovascular, biochemical and genetic measures will also be collected at different time points as part of ARCLI. These are described below and listed in Table 2.

**Table 2:** Summary of the secondary outcomes implemented in ARCLI across all time points

	Training	Baseline	3 month	6 month	12 month
**Screening**	Medical history & screen (GDS, MMSE, DRS-II) Demographic Q	Medical screen (ie BMI, MMSE, medication use)	Medical screen (ie BMI, MMSE, medication use)	Medical screen (ie BMI, MMSE, medication use)	Medical screen (ie BMI, MMSE, medication use)
**Biochemical**	Fasting Glucose & Insulin* APOE & SNP	F2 Isoprostanes Cytokines* & CRP MBA Telomere length	F2 isoprostanes Cytokines* & CRP MBA	-	F2 isoprostanes Cytokines* & CRP MBA Telomere length
**Cardiovascular**	-	Brachial BP Aortic BP carotid-femoral PWV	Brachial BP Aortic BP carotid-femoral PWV	Brachial BP Aortic BP carotid-femoral PWV	Brachial BP Aortic BP carotid-femoral PWV
**Health**	FFQ Chalder Fatigue Leeds Sleep evaluation Trait anxiety	General Health Q State anxiety Leeds Sleep Evaluation BDI-II POMS	General Health Q State anxiety Leeds Sleep Evaluation BDI-II	General Health Q State anxiety Leeds Sleep Evaluation BDI-II	FFQ General Health Q State anxiety Leeds Sleep Evaluation BDI-II
**Other**	NEO PI-R WASI				Estimate of compliance

* = Analysis performed for morning participants only

GDS = Geriatric Depression Scale, MMSE = Mini Mental State Examination, DRS = Mattis Dementia Rating Scale, Q = Questionnaire, APOE = Apolipoprotein E, SNP = Single Nucleotide Polymorphisms, WASI = *Wechsler Abbreviated Scale of Intelligence *FFQ = Food Frequency Questionnaire, NEO PI-R = NEO Personality-Inventory Revised, POMS = Profile of Mood States, BMI = Body Mass Index, CRP = C Reactive Protein, MBA = Multiple Blood Analysis, BP = Blood Pressure, PWV = Pulse Wave Velocity

### Mood, health and dietary habits

Numerous self-report questionnaires will be used to assess mood and general health. Depressive symptoms and state-trait anxiety will be measured with the Beck Depression Inventory II (BDI-II) [52] and the Spielberger State-Trait Anxiety Inventory [53] respectively. Further assessment of mood will be performed with the Profile of Mood Scales [54] and the Bond and Lader Visual Analogue Scales [55]. Throughout the trial, general health, fatigue and sleep quality will be assessed with the General Health Questionnaire [56], Chalder Fatigue Scale [57] and the Leeds Sleep Evaluation Questionnaire [58] respectively. Dietary habits will be inferred from an in-house Food Frequency Questionnaire.

### Cardiovascular

Brachial pressures, aortic pressures and carotid-femoral Pulse Wave Velocity (PWV) are not only associated with cardiovascular disease risk and mortality [59,60] but also cognitive performance and decline [61,62]. These variables can all be modified by diet and lifestyle changes [63-65] and will therefore be monitored throughout the trial as one possible mechanism by which the study supplements improve brain function. Brachial blood pressure will be measured after a 5 minute rest period using a clinically validated automated sphygmomanometer. Applanation tonometry of the radial artery will be used to estimate aortic pressures and wave reflections using a non-invasive SphygmoCor device. The same SphygmoCor device will be used to measure PWV through applanation of the carotid and femoral arteries. In addition to this assessment, cerebral and common carotid blood flow velocity and endothelial dependent vasodilation of the brachial artery may be measured on a subset of participants using transcranial Doppler and flow mediated dilation respectively.

### Biochemical

Pre-randomization, one off biochemical assessment will be conducted to measure glycated haemoglobin (HbA1c) and insulin levels. Measurement of blood glucose and insulin will provide a measure of baseline glucoregulatory efficiency and control, which may contribute to age-related cognitive decline [66]. At both baseline and selected follow-up time points, biochemical markers of oxidative stress, inflammation and safety profiling will be measured through high sensitivity C-Reactive Protein, F2-Isoprostanes, inflammatory cytokines (e.g. TNF-α, IL-2, IL-4, IL-6, IL-10 & IFN-gamma) and Liver Function Tests.

### Genetic

Blood collected pre-randomization will also be used to assess a platform of Single Nucleotide Polymorphisms thought to be related to cognitive/brain function, general health or response to treatment. This will include assessment of the APOE4 allele, which is associated with an increased risk of AD [67] and cognitive decline in normal elderly [68] but also more than 100 targeted polymorphisms including BDNF, cytokines amongst others. Genotyping will allow investigation of whether certain polymorphisms affect response to treatment or show differential relationships with the cognitive and biological variables. Telomere length (a marker of genetic damage) will also be assessed at baseline and 12 months to investigate whether any of the treatments modulate telomere shortening over the duration of the study.

### Analysis

The primary analysis will investigate the effects of treatment on all cognitive outcomes over the course of the study using Analysis of Variance (ANOVA) techniques. Other more powerful statistical techniques such as linear mixed modelling and intention to treat analysis will be considered. Similar statistical techniques will be used to investigate the effects of treatment on the secondary outcomes. Pearson's correlation coefficients will be used to investigate whether any improvements in cognition are related to improvements in other variables of interest such as biochemical, cardiovascular or mood/health factors. Correlations and regression models may be used to examine baseline associations between variables. Results will be considered statistically significant at p < 0.05 corrected for multiple cognitive factors (primary outcome variables) or p < .05 for secondary outcome variables.

Covariates such as age, gender and baseline cognitive screening measures (e.g., MMSE and Wechsler intelligence) will be adjusted for in the analyses. Results will be presented as appropriate effect sizes with a measure of precision (95% confidence intervals). Compliance to treatment will be analysed by counting each participants remaining supplements once they have completed the trial.

### Safety and Data Monitoring Committee

A data and safety committee will comprise the study GP (Prof Myers), the study nurse, the Chief Investigator (Prof Stough) and a medical officer attached to the Centre for Human Psychopharmacology but not otherwise part of ARCLI.

## Conclusions

Given our currently ageing population, research addressing the issue of cognitive decline in aged individuals is critical. This issue is further reinforced by the fact that such cognitive decline frequently precedes various forms of dementia. The proposed study will be the largest and most definitive yet assessing the role of the cognitive enhancers *Bacopa *and Pycnogenol on combined cognitive and biological measures in elderly participants. Improving the cognitive functioning of elderly citizens will have significant benefits at the societal, economic and personal levels. Cognitive capacity is strongly correlated with performance in many occupations and ameliorating the decline in fluid intelligence, memory and reasoning will allow many elderly people to continue to work and to continue to contribute to our society as they age. Coupled with the large sample size, ARCLI is one of the most definitive studies to explore the effects of *Bacopa *and Pycnogenol on cognitive ageing. Results from this study may help guide policies and preventative health practices in the elderly.

## Abbreviations

AD: Alzheimer's Disease; ANOVA: Analysis of Variance; APOE: Apolipoprotein E; ANZCTR: Australia and New Zealand Clinical trials Registry; ARCLI: Australian Research Council Longevity Intervention; BMI: Body Mass Index; BP: Blood Pressure; CDR: Cognitive Drug Research; CRP: C Reactive Protein; DRS: Mattis Dementia Rating Scale; FFQ: Food Frequency Questionnaire; GDS: Geriatric Depression Scale; IQ: Intelligence Quotient; MBA: Multiple Blood Analysis; MMSE: Mini Mental State Examination; NEO PI-R: NEO Personality-Inventory Revised; POMS: Profile of Mood States; PWV: Pulse Wave Velocity; Q: Questionnaire; RCT: Randomized, Controlled Trial; ROS: Reactive Oxygen Species; RT: Reaction Time; SNP: Single Nucleotide Polymorphisms; SUCCAB: Swinburne University Computerized Cognitive Assessment battery; WASI: Wechsler Abbreviated Scale of Intelligence; WM: Working Memory.

## Competing interests

The authors declare that they have no competing interests.

## Authors' contributions

CS and AS conceived the study. CS, AS and KC were applicants for funding. All authors were involved in designing the study and drafting the protocol. All authors read and approved the final protocol.
